# Precautionary Behavior Practices and Psychological Characteristics of COVID-19 Patients and Quarantined Persons

**DOI:** 10.3390/ijerph18116070

**Published:** 2021-06-04

**Authors:** Yubin Lee, Byung-Woo Kim, Shin-Woo Kim, Hyunjin Son, Boyoung Park, Heeyoung Lee, Myoungsoon You, Moran Ki

**Affiliations:** 1 Department of Public Health Sciences, Graduate School of Public Health, Seoul National University, Seoul 08826, Korea; ubeanee@snu.ac.kr; 2 Department of Cancer Control and Population Health, Graduate School of Cancer Science and Policy, National Cancer Center, Goyang 10408, Korea; bero@ncc.re.kr; 3 Department of Internal Medicine, School of Medicine, Kyungpook National University, Daegu 41944, Korea; ksw2kms@knu.ac.kr; 4 Department of Preventive Medicine, College of Medicine, Dong-A University, Busan 49201, Korea; hjson@dau.ac.kr; 5 Department of Preventive Medicine, College of Medicine, Hanyang University, Seoul 04763, Korea; hayejine@hanmail.net; 6 Center for Preventive and Public Health, Seoul National University Bundang Hospital, Seongnam 13620, Korea; wanderingstone@gmail.com; 7 Gyeonggi Public Health Policy Institute, Seongnam 13605, Korea

**Keywords:** COVID-19 pandemic, quarantine, isolation, public health emergency preparedness, online survey of patients and contacts

## Abstract

Background: since the coronavirus disease (COVID-19) was first reported in 2019, South Korea has enforced isolation of patients with confirmed cases of COVID-19, as well as quarantine for close contacts of individuals diagnosed with COVID-19 and persons traveling from abroad, in order to contain its spread. Precautionary behavior practices and psychological characteristics of confirmed and quarantined persons were investigated for planning pandemic recovery and preparedness. Methods: this study was conducted with 1716 confirmed patients and quarantined persons in Daegu and Busan, regions where a high number of cases were confirmed during the early stage of the COVID-19 outbreak in South Korea. We collected online survey data from 23 April to 20 May 2020, in Daegu, and 28 April to 27 May 2020, in Busan, in cooperation with Daegu and Busan Infectious Disease Control Centers and public health centers in the regions. COVID-19 symptoms, pre-cautionary behavior practices, psychological states, and the need for improvement in isolation/quarantine environments were examined using an online survey. Results: compared to patients infected with coronavirus, quarantined persons engaged in more hygiene-related behaviors (e.g., hand washing, cough etiquette, and proper mask-wearing) and social distancing. COVID-19 patients had a strong fear of stigma, while quarantined persons had a strong fear of contracting COVID-19. Study participants responded that it was necessary to provide financial support and adequate information during isolation/quarantine. Conclusions: the study highlights the importance of precautionary behavior to prevent COVID-19 infection and the need to provide support (both psychological and financial) to patients and quarantined persons, to reinforce effective communication, social solidarity, and public health emergency preparedness (PHEP) in a pandemic situation.

## 1. Introduction

Since the first report of an emerging coronavirus in Wuhan, China, in December 2019, the virus has spread rapidly worldwide [1]. In South Korea, the first confirmed case of COVID-19 occurred on 19 January 2020 [2]. In February of the same year, beginning with a cluster infection among Shincheonji Church members, the number of COVID-19 cases rose sharply in the city of Daegu and Gyeongsangbuk Province, followed by ongoing sporadic group infections occurring nationwide [2]. To date, domestic outbreaks continue to appear, and the virus continues to spread globally.

COVID-19 symptoms include fever, cough, fatigue, body aches, headache, sore throat, diarrhea, loss of taste, and loss of smell [3]. It is reported that, in most persons infected with SARS-CoV-2, the symptoms are mild or moderate, and approximately 30% of cases are asymptomatic [4,5]. In comparison to SARS and MERS, COVID-19 has a lower mortality rate, but its basic reproduction number (R_0_) is reported to be as high as 2.87 (95% CI, 2.39–3.44) [5]. As it is hard to implement pharmaceutical interventions, such as vaccines and anti-viral medications, in the pandemic, non-pharmaceutical interventions have been emphasized to prevent the spread of infection [6]. Public health authorities and experts have informed the public (and shared guidelines) on the importance of precautionary practices. A large number of countries have used interventions, such as restricting the use of public and multi-purpose facilities, prohibiting large gatherings, closing borders, and/or practicing lockdowns, although the extent of these interventions has varied across countries.

Persons confirmed with COVID-19 (or suspected of being exposed) are isolated from others and restricted in their movements in order to inhibit person-to-person transmission [7]. In South Korea, patients with moderate to critical cases are admitted to hospitals for treatment, while patients with asymptomatic or mild cases are isolated in residential treatment centers where they receive healthcare services and their symptoms are monitored. In addition, regardless of the presence or absence of symptoms, close contacts of COVID-19 patients, and anyone traveling from abroad, are required to quarantine in a residential treatment center or at home, for a period of two weeks [8].

A pandemic affects the public’s physical and mental health; these health effects were also identified during the 2019 coronavirus outbreak [9,10,11,12]. In the early stage of a pandemic, people feel fear, anxiousness, and helplessness due to the lack of information and uncertainty about the new risks (as well as fear of death) [13,14]. Anxiety regarding an emerging infectious disease can lead to suspicion and distrust of others, and people often blame those who are believed to have spread the disease [13]. Therefore, isolation and quarantine are effective at reducing the number of confirmed cases and mortality, but at the same time, they have negative psychological impacts on confirmed patients and quarantined persons [15,16]. Isolated or quarantined persons may face undesirable experiences and feelings, such as guilt, embarrassment, and social stigma, during and after the isolation or quarantine period [16,17]. They may suffer from social rejection and excessive blame, as well as fear of infection [13].

As confirmed, quarantined persons are exposed to various stresses in a COVID-19 outbreak; thus, investigating their isolation/quarantine experiences and mental health statuses is important for pandemic recovery and preparedness. Previous studies on quarantine/isolation experience assessed the mental health of members of the general public who were under mass quarantine due to COVID-19, which is different from the situation of Korea [18,19]. Studies conducted in Korea assessed the mental health status of (i) caregivers at a children’s hospital who were quarantined due to contact with a case of COVID-19 [20], and (ii) isolated patients in residential treatment centers [21]. To our knowledge, this paper is the first to report on the precautionary behavior practices and mental health of confirmed patients and quarantined persons, and identify the needs for improvement regarding isolation or quarantine during the early stage of the COVID-19 outbreak in Korea.

We investigated precautionary behavior practices (hygiene-related behavior and social distancing) for the two weeks before they had been confirmed or quarantined, since it is widely accepted that these practices may contribute to inhibiting the infection [22,23]. We investigated the psychological states of persons who experienced COVID-19-related isolation or quarantine, and the areas that needed improvement (in regards to isolation/quarantine); this is important to improve the care of persons in isolation or quarantine, to assist in their psychological recovery. This study was performed after the first wave of COVID-19 in South Korea, which occurred from February to March 2020, and was conducted with patients confirmed to have COVID-19 and quarantined persons in the regions where a high number of confirmed cases were initially reported (Daegu, Busan, Korea).

The study findings are expected to provide government organizations and healthcare professionals with basic data to improve policies that support persons who experience isolation or quarantine. An additional purpose of the current study is to increase public health emergency preparedness (PHEP) by promoting effective communication and emphasizing social solidarity during the persistent COVID-19 pandemic.

## 2. Materials and Methods

### 2.1. Data Collection

This study was conducted with patients confirmed to have COVID-19, and persons in Daegu and Busan who were isolated (or quarantined) and released from isolation/quarantine during the first wave of the COVID-19 outbreak in South Korea (February–March 2020). Daegu and Busan were the regions in South Korea where the virus spread accelerated during the early stages of the COVID-19 pandemic. As of 31 March 2020, 69.5% of all confirmed cases in the country had occurred in these two regions [24].

In this study, a confirmed case refers to an individual who tested positive on a re-verse transcription-polymerase chain reaction (RT-PCR) test and was treated at a hospital or residential treatment center. A quarantined person refers to an individual who quarantined for two weeks after being ordered to by health authorities, due to close contact or travel abroad, and who tested negative on the final test.

We used an online survey to investigate precautionary behavior practices, for two weeks before COVID-19 confirmation or quarantine, and to investigate COVID-19-related perceptions. The surveyed areas were Daegu and Busan, and all confirmed and quarantined persons who were released from quarantine at the time of the investigation were subject to investigation. However, since Daegu concentrated on the management of confirmed patients as the first outbreak, the management of isolated persons was difficult, so the investigation was excluded. The survey data were collected by sending text messages with a survey link to persons confirmed to have COVID-19, and quarantined persons, in cooperation with Daegu and Busan Infectious Disease Control Centers and public health centers in the regions. Of those, a total of 1716 (1130 patients and 586 quarantined persons) responded to the online survey. By region, in Daegu, the survey link was sent to 5626 patients between 23 April and 20 May 2020, and data were collected from 1100 individuals (19.6%). In Busan, the survey link was sent to 118 patients and 9500 quarantined persons between 28 April and 27 May 2020, and data were collected from 30 (25.4%) and 586 (6.2%) individuals, respectively. The study was approved by the IRB of the Korea National Cancer Center (NCC2020-0104). The data did not contain personally identifying information. All survey participants consented to participate in the study before responding to the survey.

### 2.2. Questionnaire

#### 2.2.1. COVID-19 Symptoms of Confirmed Patients

COVID-19-confirmed patients were asked what COVID-19 infection-related symptoms they experienced. Specifically, they were instructed to self-report the symptoms they experienced during the period of treatment or quarantine, by selecting from the following list of symptoms: fever, chills, headache, cough, phlegm, muscle aches, sore throat, difficulty breathing, loss of smell, loss of taste, nausea, indigestion, diarrhea, and others.

#### 2.2.2. Precautionary Behavior Practices for Two Weeks before Isolation/Quarantine

The degree to which individuals engaged in precautionary behavior practices for two weeks prior to quarantine or COVID-19 confirmation was assessed using 14 items. Of those, four items concerned handwashing (Q1. I always washed my hands after going to the bathroom; Q2. I always washed my hands (or used hand sanitizer) before eating; Q3. I washed my hands (or used hand sanitizer) if I thought that my hands might have been contaminated because I shook hands, touched the mask, or held a doorknob; Q4. I washed my hands when I returned home from outside), one concerned cough etiquette (Q1. I covered my mouth with tissue when coughing or coughed into my elbow), four concerned mask-wearing (Q1. I always wore a mask during hospital visit; Q2. I always wore a mask when talking with someone within a two-meter radius; Q3. I wore a mask by ensuring that the mouth and the nose are covered; Q4. I tried to avoid touching the surfaces of used masks), and five concerned person-to-person contact (Q1. I did not attend social gatherings; Q2. My working arrangements has changed (e.g., video or online conferences, working from home, flexible work arrangement, etc.); Q3. I tried to avoid eating out; Q4. I avoided mass gatherings that might bring me into contact with many people; Q5. I avoided contact with others when I had symptoms like fever and a cough). Survey participants self-reported in regard to their precautionary behavior practices, for two weeks before quarantine or COVID-19 confirmation, on a 5-point Likert scale (1 = “not at all” and 5 = “very often”). The item reliability analysis showed that Cronbach’s α coefficients were 0.844 for hand washing, 0.866 for mask-wearing, and 0.902 for person-to-person contact (Table 1).

#### 2.2.3. Perceptions of COVID-19 Infection and Psychological States of Persons Who Experienced Isolation/Quarantine

Survey participants’ perceptions of COVID-19 infection and their psychological states were assessed through seven items. Of those, three items concerned whether the respondent believed that patients were responsible for the COVID-19 infection (Q1. COVID-19 patients can prevent themselves from contracting the virus; Q2. COVID-19 patients are responsible for their own infection; Q3. It is the COVID-19 patients’ own fault that they have the disease) and four concerned fears due to the COVID-19-related situation—two items regarding fear of infection (Q1. I am afraid that I will be re-infected with COVID-19 after receiving treatment; Q2. I am afraid that I will not be fully recovered) and two regarding fear of stigma (Q1. I am afraid of being blamed because I was a confirmed patient infected with COVID-19; Q2. I am afraid that if there are confirmed cases in my area, the area will be criticized or damaged for the reason). The items were all rated on a 5-point Likert scale (1 = “not at all” and 5 = “strongly agree”). The three items regarding the attribution of COVID-19 infection were developed by the researchers in reference to Mak et al. (2006) [25].

To investigate psychological states in the COVID-19 situation, we asked about physical and mental changes (Q1. I am obsessed with searching for COVID-19 news and information; Q2. I am cautious and dubious about other people because I am afraid of getting re-infected; Q3. I feel helpless and am losing interest in what I did well before; Q4. I get more easily annoyed and upset than before; Q5. I have experienced a physical response, such as headache, indigestion, and insomnia) the participants experienced after they were confirmed with COVID-19, or received an order for quarantine, as well as disruption in daily life (Q1. How much did your daily life differ because of the COVID-19 out-break?) due to COVID-19. The items were developed by the researchers with reference to a guide by the COVID-19 Integrated Mental Health Service Team (2020). Stress due to infectious disease was assessed with five items on a 4-point-Likert scale (1 = “not at all” and 4 = “strongly agree”) [26]. Perceived daily life disruption due to COVID-19 was assessed by using one item on an 11-point scale (0 = “completely stopped” and 10 = “no change”) [27].

The item reliability analysis revealed that Cronbach’s α coefficients were 0.599 for attribution of COVID-19 infection, 0.704 for fear of infection, 0.759 for fear of stigma, and 0.816 for stress (Table 1).

#### 2.2.4. Needs of Persons Who Experienced Isolation/Quarantine

To identify the areas in which improvements were needed in the quarantine and treatment procedures, the researchers developed the six items (Q1. Early detection of the confirmed patient; Q2. Quality of the treatment of the confirmed patient; Q3. Psychological and emotional support for the confirmed patient; Q4. Financial support for the confirmed patient; Q5. Protection of human rights and privacy for the confirmed patient; Q6. Providing adequate information for the confirmed patient) on a 5-point Likert scale (1 = “not at all” and 5 = “strongly agree”). Item reliability analysis was conducted and the Cronbach’s α coefficient was 0.722 (Table 1).

### 2.3. Statistical Analysis

To examine the participants’ characteristics, they were categorized into two groups (patients and quarantined persons) on the basis of their COVID-19 experience, and frequency analysis was performed. A chi-square test (χ^2^) was performed to test for differences in the presence or absence of COVID-19-related symptoms in patients, according to sex and age group, and an independent *t*-test was performed to test for differences between the groups of patients and quarantined persons in precautionary behavior practices, psychological states, and needs. Additionally, independent t-tests and analyses of variance (ANOVA) were performed to examine differences by sex and by age group across all participants. The statistical analyses were conducted using SAS software version 9.4 (SAS Institute, Cary, NC, USA).

## 3. Results

### 3.1. Survey Participants 

A total of 1716 individuals responded to the survey, of whom 1130 (65.9%) were COVID-19-confirmed patients and 586 (34.1%) were quarantined persons. Of the total participants, 600 (35%) were male and 1116 (65%) were female. The mean age was 35.8 years. By age group, 804 (46.9%) were under 29 years of age, 297 (17.3%) were 30–39, 264 (15.4%) were 40–49, 246 (14.3) were 50–59, and 105 (6.1%) were 60 years or older. A total of 471 (27.4%) participants recently entered South Korea from abroad in 2020. Of those, 33 had confirmed cases of COVID-19 and 438 were quarantined. Thus, it was found that approximately one out of every four quarantined persons was a recent traveler from abroad. A total of 417 (24.3%) participants had received a flu vaccine after October 2019. Regarding self-reported health status at the time of the study, 1045 (60.9%) participants answered “good”, 529 (30.8%) responded “fair”, and 142 (8.3%) answered “poor” (Table 2).

### 3.2. COVID-19 Infection Symptoms

With respect to COVID-19-related symptoms in confirmed patients, 834 (73.8%) were symptomatic and 296 (26.2%) were asymptomatic. Of the symptomatic cases, 588 (78.5%) were female and 246 (64.6%) were male, showing that the proportion of symptomatic cases was higher in women than in men (*p* < 0.001). However, the proportion did not vary according to age groups.

Of the individual COVID-19 symptoms, the most common was loss of smell (38.3%), followed by loss of taste (36.5%), cough (32.7%), muscle aches (31.3%), fever (28.4%), headache (27.6%), phlegm (26.6%), sore throat (24.4%), diarrhea (22.7%), chills (21.9%), difficulty breathing (10.0%), indigestion (9.2%), and nausea (6.8%) (Table 3).

### 3.3. Precautionary Behavior Practices for Two Weeks before Isolation/Quarantine

The analysis of the scores for precautionary behavior practice, for two weeks before isolation/quarantine in patients and quarantined persons, showed that the item regarding hand washing, “I always washed my hands after going to the bathroom”, scored the highest in both patients and quarantined persons, with mean scores of 4.3 and 4.66, respectively. In comparison, the frequency of practice was lower for the item “I washed my hands (or used hand sanitizer) if I thought that my hands might have been contaminated because I shook hands, touched the mask, or held a doorknob”, with mean scores for 3.45 in patients and 4.19 for quarantined persons. Moreover, among the items concerning correct mask-wearing, “I always wore a mask during hospital visit” showed the highest practice level with mean scores of 4.12 for confirmed persons and 4.54 for quarantined persons. Of the items concerning person-to-person contact, “I avoided contact with others when I had symptoms like fever and a cough” showed the highest practice level, with mean scores of 4.12 for confirmed persons and 4.49 for quarantined persons. Overall, the precautionary behavior practice level was higher in quarantined persons than confirmed persons for all items (*p* < 0.001) (Table 4).

The analysis conducted on the difference between genders in precautionary behavior practices showed that the mean scores for hand washing and cough etiquette were higher for women than men (*p* < 0.05); in regards to mask-wearing and person-to-person contact, the results varied by item (Table 4). The age group difference was found in seven out of the total 14 items regarding precautionary behavior practices (*p* < 0.05). Of those, two items on person-to-person contact showed an opposite trend in comparison to the remaining items, which indicated higher scores as age increased (Supplementary Table S1).

### 3.4. Psychological States in Persons Who Experienced Isolation/Quarantine

The level of attribution, i.e., the extent to which the responsibility of infection was attributed to patients, was lower in patients than in quarantined persons (*p* < 0.05). Regarding situational fear, fear of COVID-19 reinfection in patients was higher than fear of COVID-19 confirmation in quarantined persons (*p* < 0.05). In contrast, fear of asymptomatic infection in quarantined persons was higher than patients fearing that they would not fully recover, but the difference was not significant (*p* = 0.074). In regard to fear of stigma, fears of criticism and disadvantage were higher in patients than in quarantined persons (*p* < 0.05) (Table 5).

Regarding stress due to infectious disease, patients reported higher stress compared to quarantined persons for all five items (*p* < 0.05). The mean score for the item concerning perceived daily life disruption was 4.26 for confirmed persons and 4.6 for quarantined persons, showing a significant difference (*p* < 0.05). An examination of the item responses classified into three groups—high level of perceived daily life disruption (scores 0–3), medium level (4–6), and low level (7–10)—showed corresponding proportions of 49%, 25.9%, and 25.1%, respectively, for confirmed persons, and 42.3%, 29.1%, 28.6%, respectively, for quarantined persons (Table 5).

The analysis of difference between genders revealed the following. The mean score for the attribution of infection was higher in men (*p* < 0.05), whereas the mean scores for fear of the situation, fear of stigma, and stress were higher in women (*p* < 0.05). In addition, the mean score of the item concerning perceived daily life disruption was 4.94 for men and 4.07 for women, showing a significant difference (*p* < 0.05) (Supplementary Table S2).

In regards to age group differences, with the exception of the age 60 or higher group, participants were more likely to answer that patients were responsible for infection as age decreased, while fear of the situation, fear of stigma, and stress increased as age increased. Regarding perceived daily life disruption, the score was lower with a decrease in age (*p* < 0.05) (Supplementary Table S2).

### 3.5. Needs of Persons Who Experienced Isolation/Quarantine

The mean score for early detection of confirmed cases and persons in quarantine was high in both patients and quarantined persons, 4.51 and 4.44, respectively, and the difference was not significant (*p* = 0.153). Whereas the need for psychological/mental support, financial support, human rights protection, and adequate information was higher in patients than in quarantined persons (*p* < 0.05). However, the need for improving health management of quarantined persons (3.92) was stronger than the need for the improvement of patient treatment (3.79) (*p* < 0.05) (Table 6).

The analysis was conducted to examine differences between genders but found no significant difference in either the need for financial support or appropriate COVID-19-related information. However, for the remaining items, the score was higher in women than in men (*p* < 0.05). With respect to between-age differences, the need for appropriate information was not significantly different among different age groups, but the remaining items showed significant age group differences (*p* < 0.05) (Supplementary Table S3).

## 4. Discussion

Participants in this study were patients with asymptomatic or mild COVID-19 cases who were treated in a hospital or residential treatment center, and persons quarantined because they had close contact with COVID-19 patients or entered the country from abroad. The participants were from Daegu and Busan, the regions in which the highest number of confirmed cases occurred during the first wave of the COVID-19 outbreak in South Korea (February–March 2020). After the new cases largely declined, we investigated COVID-19 symptoms and precautionary behavior practices (for two weeks before isolation or quarantine) and psychological states of patients and quarantined persons through a survey. Additionally, we examined the areas in which the participants felt that support would be needed during the isolation or quarantine period.

A break from daily life was the greatest change experienced by individuals due to COVID-19. This experience may have a greater significance, especially at the beginning of an infectious disease outbreak. Our study is of academic significance in that the survey investigated the isolation/quarantine experience of residents of Daegu and Busan, who experienced geographic discrimination and stigma during the first COVID-19 outbreak in South Korea, because a high number of confirmed cases occurred in these regions. Furthermore, the study has significance for the development of health policies in that lessons and implications were derived from the participants’ experiences and effort was made to identify the ways to provide other forms of support in addition to treatment.

It is expected that the study findings will help understand the isolation/quarantine experience due to COVID-19 and identify factors that contribute to improving isolation and quarantine environments.

There are a few notable findings in the study. First, 26.2% of patients confirmed to have COVID-19 were asymptomatic. The proportions of asymptomatic COVID-19 patients reported varied, depending on the timing of the study and the number of study participants [28,29]. An asymptomatic case refers to a patient who tests positive on an RT-PCR test, but does not show any COVID-19-related symptoms, such as fever or a cough, either on the day of testing or for the 14 days following [30]. Because symptoms may occur a few days after a COVID-19 test (in which case, the patient is classified as pre-symptomatic), there are limitations in estimating the proportion of asymptomatic patients based on a cross-sectional study [31]. Therefore, to avoid overestimating the proportion of asymptomatic cases, a follow-up period of approximately two weeks is required. The participants in this study were those who finished the treatment and, hence, pre-symptomatic patients were not included. The proportion of asymptomatic patients in the study was similar to the findings in a previous study, in which the proportion of asymptomatic cases was estimated by following-up with patients [4].

Second, it was found that the practice of hygiene-related behaviors and social distancing were higher in participants not infected with COVID-19 (that is, quarantined persons) than those confirmed with COVID-19. Particularly, patients and quarantined persons’ preventative behaviors differed among items concerning specific practices. For instance, the between-group difference was greater for the item “wearing a mask by ensuring that the mouth and the nose are covered” (3.91 for confirmed persons and 4.44 for quarantined persons) than for the item “wearing a mask during hospital visit” (4.12 for confirmed persons and 4.54 for quarantined persons). Additionally, the level of practicing precautionary behaviors was higher in women than in men, which is consistent with a previous study [32]. However, the difference between genders in practicing precautionary behaviors was not as great as the difference between infected and uninfected COVID-19 persons, and there was no significant between-gender difference in regard to practicing social distancing.

It was reported in the literature that, aside from sociodemographic factors, psychological factors also affect precautionary behavior practices during a pandemic [33]. In a study by Lee and You (2020), individuals who had a higher risk perception of COVID-19 and a higher efficacy of practicing precautionary behaviors practiced personal preventive behaviors and social distancing more rigorously [34]. Accordingly, it is highly likely that, compared to people infected with COVID-19, uninfected people more strongly perceived the severity of COVID-19 infection, and believed that infection could be prevented by practicing precautionary behaviors. Such a difference in perception may have resulted in the difference in precautionary behavior practices, and potentially the difference between infection and non-infection.

Third, the fear of COVID-19 showed different patterns in patients and quarantined persons. Patients were more afraid of social stigma, while quarantined persons feared COVID-19 infection more. In patients, the greatest fear was that they might be socially stigmatized due to the infection and the strongest need in response to COVID-19 was human rights protection. It is likely that their fear of stigma was influenced by the social awareness that individuals are responsible for having contracted the virus. In this study, the perception that patients were responsible for COVID-19 infection was higher in quarantined persons compared to the patients. Likewise, a survey conducted in Gyeonggi Province, South Korea, reported that there was a difference in perception on attribution of the disease between the general public and confirmed patients [35]. If a person believes that individuals have control over whether or not they become infected, he/she will perceive that patients are responsible for the illness [25,36]. The perception that patients are responsible for the cause of illness leads to negative emotions and behaviors toward patients confirmed to have COVID-19, even resulting in prejudice and discrimination [36]. Since stigma around COVID-19 infection affects all areas of patients’ lives, the government and healthcare professionals should use public communication to reduce stigma against patients confirmed to have COVID-19, while stressing the importance of precautionary behavior practices.

Whereas, in quarantined persons, the greatest fear was COVID-19 confirmation; the strongest need in response to COVID-19 was early detection of persons who should practice quarantine. Quarantined persons’ fear of a diagnosis (of infection) was also reported by Chen et al., who examined the quarantine experience of close contacts of COVID-19 patients [37]. Quarantined persons who were close contacts, not fully informed of the infectious disease, and who experienced infection-related symptoms, had a fear of infection [37,38]. The fear gradually decreased as they acquired more information on the nature of infection during quarantine and tested negative for COVID-19 [37]. However, the quarantined persons in the current study had a fear of infection, even though they did not have symptoms during quarantine and did not test positive. The finding suggests that fear of infection may be a persistent stress factor for quarantined persons regardless of the test result. Hence, central and local governments should follow-up with persons released from quarantine due to COVID-19 to understand their psychological states and support them in utilizing professional psychological intervention programs.

Finally, survey participants expressed a desire for financial support and adequate information during isolation/quarantine. Economic loss due to isolation/quarantine and insufficient information during the pandemic were identified as stress factors in another study as well [16]. If patients and quarantined persons are not guaranteed income (when they cannot work due to isolation/quarantine and afterwards), their livelihoods can be threatened. In particular, because persons with low household incomes are greatly impacted by even a temporary reduction in income, a change in income due to isolation/quarantine can significantly affect their health [39]. South Korea implemented a policy—effective as of 17 February, 2020—that workers quarantined or admitted to hospital due to COVID-19 receive paid leave from their employers, or a living allowance from the government [40]. Nevertheless, survey participants had a high level of need for financial support policy. The reason is believed to be because, in South Korea’s current financial support policy, workers who cannot work due to illness are guaranteed to receive merely the minimum level of income [40]. Accordingly, the government should develop a system to help isolated or quarantined persons smoothly return to society, such as resuming work with their employer after recovery from the infection (or after release from quarantine), and not being disadvantaged by the employer’s personnel decisions.

While isolated or quarantined, people want to have timely and trustworthy information regarding infection treatment and isolation/quarantine, and feel depression and fear if they do not have access to such information [41,42]. A great majority of survey participants responded that adequate information should be provided for COVID-19 patients and quarantined persons (92.1% and 85.1%, respectively). Approximately one-half of survey participants (55.3% of patients and 45.4% of quarantined persons) responded that they became overly obsessive about obtaining COVID-19 information after conformation of COVID-19 diagnosis or after receiving the quarantine order. Providing accurate information for isolated or quarantined persons to make health-related decisions, namely, empowering them, helps decrease a sense of helplessness and maintain good mental health during isolation/quarantine [42]. Thus, healthcare workers should explain the guidelines for isolation/quarantine and inform COVID-19 patients and quarantined persons of potential negative emotions that may be felt during isolation/quarantine so that they may better cope with the situation.

Our study shows the need for social solidarity and effective communication in the pandemic. COVID-19 patients and quarantined persons are often criticized, discriminated against in the community and at work, or ostracized because they are infected (or had contact) with confirmed patients [43]. An experience of physical and social isolation from society has psychological impacts, including depression, loneliness, frustration, and anxiety, which can persist even after a pandemic ends [43,44]. Not only does the stigma of infection affect personal health, but it is also unhelpful for infection management (from a social perspective). Due to the fear of social stigma, some people may hide the fact that they have COVID-19, avoid immediate use of healthcare services, or forgo adopting healthy behaviors [45]. Accordingly, it is important for public health authorities to provide accurate, persistent, and trustworthy information regarding COVID infections, while simultaneously stressing social solidarity.

From this point of view, our study highlights the importance of strengthening PHEP in a public health emergency, such as a pandemic. PHEP refers to “the capability of the public health and health care systems, communities, and individuals to prevent, protect against, quickly respond to, and recover from health emergencies” [46]. PHEP capabilities include conducting public health surveillance and epidemiological research, providing healthcare services, and performing non-pharmaceutical interventions (e.g., isolation and quarantine), as well as sharing accurate and efficient information, mental health promotion, and encouraging a return to normal daily life [47]. To develop PHEP capabilities, governments and private sectors, non-governmental organizations, and individuals should make continuous and concerted efforts [48].

The current study examined physical symptoms of COVID-19 and the psychological states and needs of patients confirmed to have COVID-19, as well as quarantined persons, and highlighted tolerance and solidarity as ways to cope with infection. The study has the following limitations. First, the study was conducted by using a self-report questionnaire after the isolation/quarantine period was over; thus, the findings may differ from those in an observational study. That is, survey participants may have not remembered the symptoms they had (recall bias) or responded that they practiced precautionary behaviors better than they actually did (social desirability bias). Second, the study findings did not reflect moderate–severe patient experiences. Considering that more than 40% of survey participants were between the ages of 20 and 29, whereas only 5.5% were 60 or older, survey participants seem biased toward younger people, the age group with a relatively higher proportion of mild patients. In addition, because the survey was conducted in the early stage of the COVID-19 outbreak, the level of precautionary behavior practices and the psychological states of the participants in a study conducted at a different time may differ, in accordance with the changes or stages of the public health emergency in South Korea.

## 5. Conclusions

Our findings on precautionary behavior practices emphasize the importance of hygiene-related behavior and social distancing to prevent COVID-19 infection. Compared to confirmed persons, quarantined persons showed better performance in hand washing, cough etiquette, proper mask-wearing, and social distancing. In addition, our findings suggest ways to improve the policies supporting persons isolated or quarantined due to COVID-19. In the present study, COVID-19 patients showed a strong fear of stigma, and quarantined persons had a strong fear of contracting COVID-19. Since stress can persist afterwards, the mental health of these individuals should be evaluated through a follow-up and they should be provided with opportunities to participate in counseling intervention programs. Individuals should be fully informed and financially supported during isolation or quarantine. The results of the present study emphasize the need for social and financial support for patients and quarantined persons, as well as health communication concerning precautionary behavior practices and anti-stigma and social solidity awareness during a public health emergency.

## Figures and Tables

**Table 1:** Contents of the questionnaire.

Classification	Questionnaires (30)	Scale	Cronbach’s α		
			Confirmed	Quarantined	Total
Symptoms *	1	Binary	n/a	−	n/a
Hand washing	4	Likert 5 points	0.831	0.823	0.844
Coughing behavior	1		n/a	n/a	n/a
Mask-wearing	4		0.888	0.856	0.886
Person-to-person contact	5		0.906	0.874	0.902
Attribution of infection	3	Likert 5 points	0.538	0.591	0.599
Fear of situation	2		0.713	0.704	0.704
Fear of stigma	2		0.759	0.711	0.759
Stress	5	Likert 4 points	0.821	0.784	0.816
Perceived daily life disruption	1	0 (completely stopped) to 10 (no change)	n/a	n/a	n/a
Needs	6	Likert 5 points	0.680	0.769	0.722

* Only confirmed patients check all symptoms at onset of infection; n/a: Questionnaires are Not Applicable.

**Table 2:** Characteristics of survey participants *.

	COVID-19 Experience		Total (n = 1716)
	Confirmed (*n* = 1130)	Quarantined (*n* = 586)	
**Sex**			
Male	381 (33.7)	219 (37.4)	600 (35.0)
Female	749 (66.3)	367 (62.6)	1116 (65.0)
**Age group**			
≤29	519 (45.9)	285 (48.6)	804 (46.9)
30–39	170 (15.0)	127 (21.7)	297 (17.3)
40–49	177 (15.7)	87 (14.9)	264 (15.4)
50–59	202 (17.9)	44 (7.5)	246 (14.3)
≥60	62 (5.5)	43 (7.3)	105 (6.1)
**Travel or visit abroad in 2020**			
No	1097 (97.1)	148 (25.3)	1245 (72.6)
Yes	33 (2.9)	438 (74.7)	471 (27.4)
**Flu vaccination since October 2019**			
No	875 (77.4)	424 (72.4)	1299 (75.7)
Yes	255 (22.6)	162 (27.6)	417 (24.3)
**Health status (Self-reported)**			
Bad	129 (11.4)	13 (2.2)	142 (8.3)
Moderate	384 (34.0)	145 (24.7)	529 (30.8)
Good	617 (54.6)	428 (73.0)	1045 (60.9)

* Mean age (standard deviation) was 36.4 (13.4) for confirmed patients and 34.8 (13.0) for quarantined persons.

**Table 3:** Symptoms of infection in confirmed COVID-19 patients (*n* = 1130).

	COVID-19 Symptoms		*p*-Value
	Yes	No	
**Total, *n* (%)**	834 (73.8)	296 (26.2)	
**Sex**			
Male	246 (64.6)	135 (35.4)	<0.001
Female	588 (78.5)	296 (21.5)	
**Age Group (Mean = 35.8)**			
≤29	371 (71.5)	148 (28.5)	0.131
30–39	134 (78.8)	36 (21.2)	
40–49	140 (79.1)	37 (20.9)	
50–59	146 (72.3)	56 (27.7)	
≥60	43 (69.4)	19 (30.6)	
Reported Symptoms *			
Fever	321 (28.4)	809 (71.6)	n/a
Chills	247 (21.9)	883 (78.1)	
Headache	312 (27.6)	818 (72.4)	
Cough	369 (32.7)	761 (67.3)	
Phlegm	301 (26.6)	829 (73.4)	
Muscle pain	354 (31.3)	776 (68.7)	
Sore throat	276 (24.4)	854 (75.6)	
Difficulty breathing	113 (10.0)	1017 (90.0)	
Cannot smell	433 (38.3)	697 (61.7)	
Cannot taste	413 (36.5)	717 (63.5)	
Nausea, Vomiting	77 (6.8)	1053 (93.2)	
Indigestion	104 (9.2)	1026 (90.8)	
Diarrhea	257 (22.7)	873 (77.3)	
Other symptoms	74 (6.5)	1056 (93.5)	

* Including 765 duplicate respondents; n/a: Questionnaires are Not Applicable.

**Table 4:** Precautionary behavioral survey response results *.

Questionnaire	Confirmed	Quarantined	*p*-Value
	Mean (SD)	Mean (SD)	
**Hand washing (4)**			
I always washed my hands after going to the bathroom.	4.30 (0.86)	4.66 (0.59)	<0.001
I always washed my hands (or used hand sanitizer) before eating.	3.75 (1.11)	4.29 (0.91)	<0.001
I washed my hands (or used hand sanitizer) if I thought that my hands might have been contaminated because I shook hands, touched the mask, or held a doorknob.	3.45 (1.22)	4.19 (0.99)	<0.001
I washed my hands when I returned home from outside.	4.15 (0.98)	4.61 (0.69)	<0.001
**Coughing behavior (1)**			
I covered my mouth with tissue when coughing or coughed into my elbow.	4.07 (1.02)	4.55 (0.71)	<0.001
**Mask-wearing (4)**			
I always wore a mask during hospital visit.	4.12 (1.15)	4.54 (0.90)	<0.001
I always wore a mask when talking with someone within a two-meter radius.	3.52 (1.30)	4.06 (1.13)	<0.001
I wore a mask by ensuring that the mouth and the nose are covered.	3.91 (1.19)	4.44 (0.91)	<0.001
I tried to avoid touching the surfaces of used masks.	3.46 (1.21)	3.94 (1.07)	<0.001
**Person-to-person contact (5)**			
I did not attend social gatherings.	3.74 (1.34)	4.18 (1.05)	<0.001
My working arrangements has changed (e.g., video or online conferences, working from home, flexible work arrangement, etc.).	3.14 (1.54)	3.83 (1.14)	<0.001
I tried to avoid eating out.	3.86 (1.27)	4.32 (0.97)	<0.001
I avoided mass gatherings that might bring me into contact with many people.	3.75 (1.31)	4.29 (0.96)	<0.001
I avoided contact with others when I had symptoms like fever and a cough.	4.21 (0.99)	4.49 (0.80)	<0.001

* For a period of two weeks before confirmation/being quarantined.

**Table 5:** Psychological survey response results.

Questionnaire	Confirmed	Quarantined	*p*-Value
	Mean (SD)	Mean (SD)	
**Attribution of infection (3)**			
COVID-19 patients can prevent themselves from contracting the virus.	2.45 (1.14)	2.82 (1.19)	<0.001
COVID-19 patients are responsible for their own infection.	2.09 (0.97)	2.82 (0.97)	<0.001
It is the COVID-19 patients’ own fault that they have the disease.	2.25 (1.06)	2.87 (0.9)	<0.001
**Fear of situation (2)**			
I am afraid that I will be re-infected with COVID-19 after receiving treatment. * I am afraid that I will be confirmed as infected with COVID-19.	3.68 (1.1)	3.46 (1.05)	<0.001
I am afraid that I will not be fully recovered. * I am afraid of being an asymptomatic infected patient.	2.9 (1.28)	3.01 (1.16)	0.074
Fear of stigma (2)			
I am afraid of being blamed because I was a confirmed patient infected with COVID-19. * I am afraid of being blamed because I was quarantined.	3.79 (1.11)	2.85 (1.32)	<0.001
I am afraid that if there are confirmed cases in my area, the area will be criticized or damaged for the reason.	3.44 (1.17)	2.91 (1.17)	<0.001
**Stress (5)**			
I am obsessed with searching for COVID-19 news and information.	2.56 (0.95)	2.34 (0.86)	<0.001
I am cautious and dubious about other people because I am afraid of getting re-infected. * I am cautious and dubious about other people because I am afraid of getting infected.	2.66 (0.84)	2.39 (0.82)	<0.001
I feel helpless and am losing interest in what I did well before.	2.47 (0.96)	2.1 (0.93)	<0.001
I get more easily annoyed and upset than before.	2.21 (0.93)	1.93 (0.91)	<0.001
I have experienced a physical response, such as headache, indigestion, and insomnia.	2.27 (1.01)	1.96 (0.96)	<0.001
**Perceived daily life disruption due to COVID-19 out-break**			
How much did your daily life differ because of the COVID-19 outbreak?	4.26 (2.83)	4.6 (2.81)	0.018

* For persons in quarantine due to COVID-19.

**Table 6:** Needs for confirmed patients and quarantined persons.

Questionnaire	Confirmed	Quarantined	*p*-Value
	Mean (SD)	Mean (SD)	
**Needs (6)**			
Early detection of the confirmed patient. * Early detection of the subject of quarantine.	4.51 (0.82)	4.44 (0.9)	0.153
Quality of the treatment of the confirmed patient. * Level of health management of the quarantined person.	3.79 (1.02)	3.92 (0.94)	0.009
Psychological and emotional support for the confirmed patient. * Psychological and emotional support for the quarantined person.	4.04 (0.94)	3.9 (0.99)	0.005
Financial support for the confirmed patient. * Financial support for the quarantined person.	4.59 (0.66)	4.06 (0.91)	<0.001
Protection of human rights and privacy for the confirmed patient.Protection of human rights and privacy for the quarantined person.	4.76 (0.58)	4.01 (1.0)	<0.001
Providing adequate information for the confirmed patient. * Providing adequate information for the quarantined person.	4.62 (0.65)	4.38 (0.84)	<0.001

* For persons in quarantine due to COVID-19.

## Data Availability

The data presented in this study are available on reasonable request from the corresponding author, and only after approval by the medical ethical committee. The data are not publicly available due to ethical restrictions.

## Supplementary Materials

The following are available online at https://www.mdpi.com/article/10.3390/ijerph18116070/s1, Supplementary Table S1. Precautionary behavioral survey response results, by sex and age. Supplementary Table S2. Psychological survey response results, by sex and age. Supplementary Table S3. Needs for confirmed patients and quarantined persons, by sex and age. Supplementary Table S4. Precautionary behavioral survey response results in persons quarantined, by contact or abroad *. Supplementary Table S5. Psychological survey response results in persons quarantined, by contact or abroad. Supplementary Table S6. Needs for quarantined persons, by contact or abroad.
