# Supplementary material for: Precautionary Behavior Practices and Psychological Characteristics of COVID-19 Patients and Quarantined Persons
Source: Int J Environ Res Public Health. 2021 Jun 4;18(11):6070. doi: 10.3390/ijerph18116070 (PMC8200059; doi:10.3390/ijerph18116070)
Supplement: Supplementary file 1 [file ijerph-18-06070-s001.zip › ijerph-1236106-supplementary.pdf]

International Journal of
Environmental Research and Public Health

MDPI

Supplementary Table S1. Precautionary behavioral survey response results, by sex and age group *.

Questionnaire	Sex		*p*-Value	Age Group					*p*-Value
	Male (*n* = 600)	Female (*n* = 1116)		≤29 (*n* = 804)	30–39 (*n* = 297)	40–49 (*n* = 264)	50–59 (*n* = 246)	≥60 (*n* = 105)	
**Handwashing (4)**									
I always washed my hands after going to the bathroom.	4.21 (0.86)	4.54 (0.74)	<0.001	4.46 (0.8)	4.55 (0.73)	4.44 (0.78)	4.25 (0.84)	4.22 (0.8)	<0.001
I always washed my hands (or used hand sanitizer) before eating.	3.78 (1.09)	4.02 (1.06)	<0.001	3.92 (1.09)	4.03 (1.08)	3.96 (1.07)	3.83 (1.03)	3.92 (0.99)	0.297
I washed my hands (or used hand sanitizer) if I thought that my hands might have been contaminated because I shook hands, touched the mask, or held a doorknob.	3.59 (1.18)	3.77 (1.2)	0.003	3.72 (1.2)	3.84 (1.2)	3.62 (1.25)	3.58 (1.2)	3.72 (1.08)	0.111
I washed my hands when I returned home from outside.	4.11 (0.96)	4.41 (0.87)	<0.001	4.37 (0.92)	4.44 (0.84)	4.29 (0.89)	4.04 (0.97)	4.1 (0.89)	<0.001
**Coughing behavior (1)**									
I covered my mouth with tissue when coughing or coughed into my elbow.	4.11 (1.0)	4.3 (0.92)	<0.001	4.33 (0.9)	4.39 (0.86)	4.08 (1.08)	3.99 (1.0)	4.0 (0.98)	<0.001
**Mask-wearing (4)**									
I always wore a mask during hospital visit.	4.14 (1.11)	4.32 (1.08)	0.001	4.34 (1.05)	4.33 (1.12)	4.21 (1.16)	4.07 (1.09)	4.09 (1.1)	0.003
I always wore a mask when talking with someone within a two-meter radius.	3.67 (1.27)	3.72 (1.27)	0.416	3.68 (1.29)	3.79 (1.29)	3.71 (1.3)	3.61 (1.19)	3.85 (1.16)	0.341
I wore a mask by ensuring that the mouth and the nose are covered.	4.01 (1.14)	4.13 (1.12)	0.03	4.16 (1.12)	4.17 (1.12)	4.02 (1.19)	3.89 (1.1)	3.98 (1.13)	0.008
I tried to avoid touching the surfaces of used masks.	3.58 (1.2)	3.65 (1.17)	0.258	3.62 (1.21)	3.67 (1.18)	3.63 (1.21)	3.55 (1.09)	3.64 (1.12)	0.852
**Person-to-person contact (5)**									
I did not attend social gatherings.	3.9 (1.25)	3.89 (1.28)	0.85	3.81 (1.33)	3.99 (1.19)	3.98 (1.21)	3.83 (1.25)	4.16 (1.08)	0.019
My working arrangements has changed. (e.g., video or online conferences, working from home, flexible work arrangement, etc.)	3.35 (1.47)	3.39 (1.56)	0.622	3.46 (1.56)	3.29 (1.53)	3.34 (1.51)	3.12 (1.5)	3.7 (1.29)	0.005
I tried to avoid eating out.	3.95 (1.22)	4.05 (1.18)	0.079	4.01 (1.22)	4.01 (1.19)	4.09 (1.17)	3.93 (1.16)	4.09 (1.13)	0.612
I avoided mass gatherings that might bring me into contact with many people.	3.89 (1.22)	3.96 (1.24)	0.27	3.89 (1.3)	3.93 (1.22)	3.99 (1.18)	3.93 (1.16)	4.14 (1.0)	0.32
I avoided contact with others when I had symptoms like fever and a cough.	4.23 (1.01)	4.35 (0.9)	0.019	4.28 (0.95)	4.36 (0.92)	4.34 (0.94)	4.26 (0.92)	4.34 (0.93)	0.595

* For a period of two weeks before confirmation/being quarantined.

Supplementary Table S2. Psychological survey response results, by sex and age group.

Questionnaire	Sex		*p*-Value	Age Group					*p*-Value
	Male (*n* = 600)	Female (*n* = 1116)		≤29 (*n* = 804)	30–39 (*n* = 297)	40–49 (*n* = 264)	50–59 (*n* = 246)	≥60 (*n* = 105)	
**Attribution of infection (3)**									
COVID-19 patients can prevent themselves from contracting the virus.	2.76 (1.24)	2.48 (1.12)	<0.001	2.69 (1.16)	2.56 (1.2)	2.41 (1.11)	2.35 (1.12)	2.71 (1.25)	<0.001
COVID-19 patients are responsible for their own infection.	2.53 (1.09)	2.24 (0.98)	<0.001	2.46 (1.01)	2.42 (1.04)	2.14 (0.99)	1.97 (0.95)	2.54 (1.13)	<0.001
It is the COVID-19 patients' own fault that they have the disease.	2.65 (1.06)	2.36 (1.02)	<0.001	2.48 (0.98)	2.45 (1.03)	2.36 (1.03)	2.39 (1.2)	2.82 (1.13)	0.003
**Fear of situation (2)**									
I am afraid that I will be re-infected with COVID-19 after receiving treatment. * I am afraid that I will be confirmed as infected with COVID-19.	3.28 (1.16)	3.78 (1.0)	<0.001	3.53 (1.11)	3.68 (1.11)	3.59 (1.02)	3.77 (1.02)	3.65 (1.07)	0.023
I am afraid that I will not be fully recovered. * I am afraid of being an asymptomatic infected patient.	2.6 (1.2)	3.12 (1.23)	<0.001	2.92 (1.27)	3.08 (1.3)	2.93 (1.18)	2.91 (1.17)	2.7 (1.11)	0.086
**Fear of stigma (2)**									

Questionnaire	Sex		p-Value	Age Group					p-Value
	Male (n = 600)	Female (n = 1116)		≤29 (n = 804)	30–39 (n = 297)	40–49 (n = 264)	50–59 (n = 246)	≥60 (n = 105)	
I am afraid of being blamed because I was a confirmed patient infected with COVID-19. * I am afraid of being blamed because I was quarantined.	3.18 (1.33)	3.63 (1.2)	<0.001	3.35 (1.31)	3.59 (1.29)	3.5 (1.21)	3.73 (1.12)	3.34 (1.18)	<0.001
I am afraid that if there are confirmed cases in my area, the area will be criticized or damaged for the reason.	3.0(1.27)	3.4 (1.13)	<0.001	3.08 (1.23)	3.41 (1.16)	3.37 (1.16)	3.54 (1.08)	3.21 (1.17)	<0.001
**Stress (5)**									
I am obsessed with searching for COVID-19 news and information.	2.33 (0.89)	2.58 (0.93)	<0.001	2.26 (0.93)	2.52 (0.94)	2.71 (0.85)	2.84 (0.82)	2.73 (0.81)	<0.001
I am cautious and dubious about other people because I am afraid of getting re-infected. * I am cautious and dubious about other people because I am afraid of getting infected.	2.38 (0.84)	2.67 (0.83)	<0.001	2.38 (0.87)	2.62 (0.8)	2.69 (0.81)	2.87 (0.73)	2.76 (0.73)	<0.001
I feel helpless and am losing interest in what I did well before.	2.15 (0.93)	2.44 (0.97)	<0.001	2.24 (0.98)	2.35 (0.99)	2.44 (0.91)	2.55 (0.93)	2.31 (0.96)	<0.001
I get more easily annoyed and upset than before.	1.99 (0.89)	2.18 (0.95)	<0.001	2.06 (0.95)	2.14 (0.93)	2.14 (0.91)	2.24 (0.9)	2.09 (0.94)	0.125
I have experienced a physical response, such as headache, indigestion, and insomnia.	1.86 (0.91)	2.32 (1.01)	<0.001	2.09 (1.03)	2.09 (0.99)	2.32 (1.0)	2.28 (0.94)	2.19 (0.86)	0.003
**Perceived daily life disruption due to COVID-19 out-break.**									
How much did your daily life differ because of the COVID-19 outbreak?	4.94 (2.87)	4.07 (2.76)	<0.001	4.07 (2.73)	4.31 (2.78)	4.6 (2.81)	4.83 (3.01)	5.29 (2.98)	<0.001

* For persons in quarantine due to COVID-19.

Supplementary Table S3. Needs for confirmed patients and quarantined persons, by sex and age group.

Questionnaire	Sex		p-Value	Age Group					p-Value
	Male (n = 600)	Female (n = 1116)		≤29 (n = 804)	30–39 (n = 297)	40–49 (n = 264)	50–59 (n = 246)	≥60 (n = 105)	
**Needs (6)**									
Early detection of the confirmed patient. * Early detection of the subject of quarantine.	4.41 (0.92)	4.52 (0.8)	0.013	4.39 (0.85)	4.47 (0.88)	4.63 (0.8)	4.63 (0.84)	4.57 (0.85)	<0.001
Quality of the treatment of the confirmed patient. * Level of health management of the quarantined person.	3.77 (1.03)	3.88 (0.98)	0.026	3.72 (0.97)	3.9 (0.92)	3.88 (1.03)	4.04 (1.04)	3.95 (1.06)	<0.001
Psychological and emotional support for the confirmed patient. * Psychological and emotional support for the quarantined person.	3.9 (1.01)	4.04 (0.92)	0.005	3.9 (0.98)	4.05 (0.89)	4.15 (0.91)	4.16 (0.94)	3.8 (0.99)	<0.001
Financial support for the confirmed patient. * Financial support for the quarantined person.	4.38 (0.82)	4.42 (0.78)	0.241	4.36 (0.81)	4.4 (0.78)	4.49 (0.76)	4.63 (0.67)	4.11 (0.93)	<0.001
Protection of human rights and privacy for the confirmed patient. * Protection of human rights and privacy for the quarantined person.	4.4 (0.91)	4.56 (0.78)	<0.001	4.45 (0.89)	4.47 (0.82)	4.61 (0.72)	4.67 (0.68)	4.36 (0.92)	<0.001
Providing adequate information for the confirmed patient. * Providing adequate information for the quarantined person.	4.5 (0.73)	4.55 (0.73)	0.172	4.51 (0.75)	4.55 (0.7)	4.58 (0.72)	4.61 (0.68)	4.42 (0.76)	0.115

* For persons in quarantine due to COVID-19.

Supplementary Table S4. Precautionary behavioral survey response results in persons quarantined, by contact or abroad *.

Questionnaire	Quarantined (n = 586) Contact (n = 177)	Abroad (n = 409)	p-Value
**Handwashing (4)**			
I always washed my hands after going to the bathroom.	4.54 (0.7)	4.72 (0.53)	0.003
I always washed my hands (or used hand sanitizer) before eating.	4.13 (0.97)	4.35 (0.87)	0.006
I washed my hands (or used hand sanitizer) if I thought that my hands might have been contaminated because I shook hands, touched the mask, or held a doorknob.	3.9 (1.07)	4.31 (0.92)	<0.001
I washed my hands when I returned home from outside.	4.43 (0.81)	4.69 (0.62)	<0.001
**Coughing behavior (1)**			
I covered my mouth with tissue when coughing or coughed into my elbow.	4.43 (0.84)	4.6 (0.64)	0.017
**Mask-wearing (4)**			
I always wore a mask during hospital visit.	4.33 (1.31)	4.63 (0.77)	0.001
I always wore a mask when talking with someone within a two-meter radius.	3.75 (1.21)	4.19 (1.06)	<0.001
I wore a mask by ensuring that the mouth and the nose are covered.	4.28 (1.02)	4.51 (0.85)	0.01
I tried to avoid touching the surfaces of used masks.	3.88 (1.05)	3.97 (1.08)	0.724
**Person-to-person contact (5)**			
I did not attend social gatherings.	3.8 (1.24)	4.35 (0.92)	<0.001
My working arrangements has changed. (e.g., video or online conferences, working from home, flexible work arrangement, etc.).	2.93 (1.48)	4.22 (1.18)	<0.001
I tried to avoid eating out.	3.9 (1.12)	4.5 (0.84)	<0.001
I avoided mass gatherings that might bring me into contact with many people.	3.84 (1.18)	4.48 (0.77)	<0.001
I avoided contact with others when I had symptoms like fever and a cough.	4.27 (0.96)	4.59 (0.7)	<0.001

* For a period of two weeks before being quarantined.

Supplementary Table S5. Psychological survey response results in persons quarantined, by contact or abroad.

Questionnaire	Quarantined (n = 586) Contact (n = 177)	Abroad (n = 409)	p-Value
**Attribution of infection (3)**			
COVID-19 patients can prevent themselves from contracting the virus.	2.64 (1.16)	2.9 (1.2)	0.017
COVID-19 patients are responsible for their own infection.	2.57 (0.96)	2.92 (0.95)	<0.001
It is the COVID-19 patients' own fault that they have the disease.	2.8 (0.92)	2.89 (0.89)	0.223
**Fear of situation (2)**			
I am afraid that I will be confirmed as infected with COVID-19.	3.59 (0.99)	3.4 (1.07)	0.039
I am afraid of being an asymptomatic infected patient.	3.05 (1.19)	2.99 (1.15)	0.546
**Fear of stigma (2)**			
I am afraid of being blamed because I was quarantined.	3.26 (1.33)	2.67 (1.28)	<0.001
I am afraid that if there are confirmed cases in my area, the area will be criticized or damaged for the reason.	3.15 (1.16)	2.8 (1.16)	<0.001
**Stress (5)**			
I am obsessed with searching for COVID-19 news and information.	2.53 (0.83)	2.26 (0.86)	<0.001
I am cautious and dubious about other people because I am afraid of getting infected.	2.5 (0.75)	2.35 (0.84)	0.042
I feel helpless and am losing interest in what I did well before.	2.16 (0.93)	2.07 (0.93)	0.284
I get more easily annoyed and upset than before.	2.09 (0.92)	1.87 (0.9)	0.006
I have experienced a physical response, such as headache, indigestion, and insomnia.	1.99 (0.94)	1.94 (0.97)	0.521
**Perceived daily life disruption due to COVID-19 out-break.**			
How much did your daily life differ because of the COVID-19 outbreak?	4.98 (2.71)	4.44 (2.85)	0.03

Supplementary Table S6. Needs for quarantined persons, by contact or abroad.

Questionnaire	Quarantined (n = 586)		p-Value
	Contact (n = 177)	Abroad (n = 409)	
**Needs (6)**			
Early detection of the subject of quarantine.	4.5 (0.87)	4.42 (0.9)	0.293
Level of health management of the quarantined person.	4.09 (0.83)	3.85 (0.97)	0.002
Psychological and emotional support for the quarantined person.	4.09 (0.89)	3.82 (1.02)	0.001
Financial support for the quarantined person.	4.2 (0.84)	4.0 (0.94)	0.013
Protection of human rights and privacy for the quarantined person.	4.29 (0.84)	3.88 (1.03)	<0.001
Providing adequate information for the quarantined person.	4.5 (0.72)	4.33 (0.88)	0.015
